# A Web-Based Risk-Reframing Intervention to Influence Early Childhood Educators’ Attitudes and Supportive Behaviors Toward Outdoor Play: Protocol for the OutsidePlay Study Randomized Controlled Trial

**DOI:** 10.2196/31041

**Published:** 2021-11-18

**Authors:** Mariana Brussoni, Christina S Han, John Jacob, Fritha Munday, Megan Zeni, Melanie Walters, Tina Cheng, Amy Schneeberg, Emily Fox, Eva Oberle

**Affiliations:** 1 Department of Pediatrics University of British Columbia Vancouver, BC Canada; 2 School of Population and Public Health University of British Columbia Vancouver, BC Canada; 3 British Columbia Injury Research & Prevention Unit British Columbia Children’s Hospital Research Institute Vancouver, BC Canada; 4 Human Early Learning Partnership University of British Columbia Vancouver, BC Canada; 5 Faculty of Education University of British Columbia Vancouver, BC Canada; 6 Child Care Services University of British Columbia Vancouver, BC Canada

**Keywords:** early years, risky play, teacher, childcare, early learning, risk perception, outdoor play

## Abstract

**Background:**

Early learning and childcare centers (ELCCs) can offer young children critical opportunities for quality outdoor play. There are multiple actual and perceived barriers to outdoor play at ELCCs, ranging from safety fears and lack of familiarity with supporting play outdoors to challenges around diverse perspectives on outdoor play among early childhood educators (ECEs), administrators, licensing officers, and parents.

**Objective:**

Our study objective is to develop and evaluate a web-based intervention that influences ECEs’ and ELCC administrators’ perceptions and practices in support of children’s outdoor play at ELCCs.

**Methods:**

The development of the fully automated, open-access, web-based intervention was guided by the intervention mapping process. We first completed a needs assessment through focus groups of ECEs, ELCC administrators, and licensing officers. We identified key issues, needs, and challenges; opportunities to influence behavior change; and intervention outcomes and objectives. This enabled us to develop design objectives and identify features of the OutsidePlay web-based intervention that are central to addressing the issues, needs, and challenges of ECEs and ELCC administrators. We used social cognitive theory and behavior change techniques to select methods, applications, and technology to deliver the intervention. We will use a two-parallel-group randomized controlled trial (RCT) design to evaluate the efficacy of the intervention. We will recruit 324 ECEs and ELCC administrators through a variety of web-based means, including Facebook advertisements and mass emails through our partner networks. The RCT study will be a purely web-based trial where outcomes will be self-assessed through questionnaires. The RCT participants will be randomized into the intervention group or the control group. The control group participants will read the Position Statement on Active Outdoor Play.

**Results:**

The primary outcome is increased tolerance of risk in children’s play, as measured by the Teacher Tolerance of Risk in Play Scale. The secondary outcome is self-reported attainment of a self-developed behavior change goal. We will use mixed effects models to test the hypothesis that there will be a difference between the intervention and control groups with respect to tolerance of risk in children’s play. Differences in goal attainment will be tested using logistic regression analysis.

**Conclusions:**

The OutsidePlay web-based intervention guides users through a personalized journey that is split into 3 chapters. An effective intervention that addresses the barriers to outdoor play in ELCC settings has the potential to improve children’s access to outdoor play and support high-quality early childhood education.

**Trial Registration:**

ClinicalTrials.gov NCT04624932; https://clinicaltrials.gov/ct2/show/NCT04624932

**International Registered Report Identifier (IRRID):**

DERR1-10.2196/31041

## Introduction

### Background

Outdoor play and its embedded risk-taking is crucial for children’s physical, social, emotional, and intellectual development [1,2]. Playing outdoors can enhance children’s self-confidence, social connectedness, physical activity, and risk management [1-3]. The United Nations Convention on the Rights of the Child codifies the importance of children’s right to play and accessibility to adequate spaces for outdoor play [4]. Generational declines in outdoor play are trending internationally [5-8]. The consequent increases in sedentary behavior may be associated with negative health consequences such as declines in Canadian children’s mental health [2] and an increase in obesity rates [9].

Declines in outdoor play are associated with a variety of societal factors such as changes in technology and increases in screen time; access to, and quality of, outdoor play spaces; parenting ideals that prioritize children’s achievement; and surveillance [8]. A main barrier relates to caregivers’ safety concerns and apprehensions of children’s risk-taking in outdoor play, which negatively affects children’s play opportunities and, subsequently, their development [2,5,6]. This includes limits in the home environment imposed by parents, restrictive policies that constrict play-space design and play behaviors in public spaces, and limitations on children’s play time and opportunities at schools and early learning and childcare centers (ELCCs). These educational environments can be critical venues to increase outdoor play opportunities and support equitable access to high-quality play because they are spaces where children spend most of their waking hours.

The importance of outdoor play became acute during the COVID-19 pandemic and its associated restrictions. In many countries, including Canada, ELCCs were closed for several weeks before reopening with COVID-19 safety protocols in place. The closures and restrictions led to significant changes in daily life for children and families, including how Canadian children engage in play and recreation. During this time of physical distancing and behavior restrictions, there were significant declines in time spent outdoors and in outdoor play among children and youth in Canada [10]. This worrying trend can compromise children’s mental health and disease resistance because play is a critical outlet for children’s stress management, and time spent outdoors playing helps boost immunity through physical activity and access to vitamin D and also supports well-being [6,11]. Children with access to friends and play reported greater well-being during the pandemic [12].

ELCCs can be important allies in supporting early childhood development. Research clearly indicates that investments in early childhood education have significant and far-reaching impacts throughout the lifespan and, importantly, can help reduce inequality, mitigating the effects of early childhood disadvantage [13]. In Canada, as well as in many other Western countries, most parents rely on ELCCs for childcare. Almost half (46%) of Canadian parents reported using childcare in 2011, with up to 86% of these parents using childcare on a regular basis [11]. Children often spend >30 hours per week in childcare, and for some children, this might be their main opportunity for outdoor play [14]. However, ELCC pedagogies are not always explicit on the importance of outdoor play, and ELCCs vary widely on their provision and quality of outdoor play opportunities [15]. Early childhood educators (ECEs) report struggles in providing stimulating outdoor play. Actual and perceived barriers include ECEs’ and administrators’ safety fears, liability concerns, and limited knowledge on the importance of outdoor play [15,16], which can result in risk aversion, restrictive rules, and a lack of engaging play spaces [16-19]. The COVID-19 pandemic has heightened challenges as well as the importance of supporting children’s outdoor time [10,20,21]. Guidelines in many jurisdictions encourage maximum outdoor time as an effective infection prevention strategy [22]. Many ELCCs are struggling with implementing these recommendations because pre-existing barriers such as parental safety concerns [23] have endured, if not intensified. The need to support a shift in ELCC practice to encourage outdoor play is clear, as is the need for an intervention that addresses the identified barriers and challenges.

Previously, we developed the OutsidePlay intervention to support parents of children aged 6-12 years to reframe their perceptions of the risks their children faced in outdoor play and to plan for a change in parenting behavior to support their children’s outdoor play [24]. We developed 2 versions of the intervention: an in-person version and a web-based version. Both were underpinned by social cognitive theory (SCT), incorporating behavior change techniques (BCTs), and took users through a personalized journey whereby they proceeded through a series of self-reflection questions and choose-your-own-adventure video scenarios to ultimately develop a plan for change. We conducted a randomized controlled trial (RCT) with 451 mothers, examining the efficacy of both versions of the intervention. The results indicated that the OutsidePlay intervention was effective in increasing mothers’ tolerance of risk in children’s play at 1 week and 3 months after the intervention, whereas an in-person workshop only indicated significant effects at 1 week [25]. The efficacy of the OutsidePlay web-based intervention for parents provided support for developing an intervention for ECEs to support outdoor play at ELCCs.

### Objective

Our study objective is to develop and evaluate a web-based intervention to influence ECEs’ and administrators’ perceptions and practices in support of children’s outdoor play within ELCCs. This paper outlines the intervention mapping (IM) process, a protocol that we followed to develop and test the intervention.

## Methods

### Study Design

IM guided the planning, development, and evaluation of the fully automated and open-access web-based intervention [26]. IM has proven effective for the development of many web-based interventions [27,28]. Furthermore, this strategy supports a theory-based approach that focuses on understanding and accommodating the needs and perspectives of the end users at all stages of the design. IM involves 6 steps, which are outlined in the context of this study in Textbox 1.

**Overview of intervention-mapping approach to developing the OutsidePlay web-based intervention for early learning and childcare centers.**

**The 6 steps involved in the intervention-mapping approach**

- Step 1: Understanding the problem
  Established key partnerships and worked with study partners. Our study partners included the coauthors, each of whom brought unique disciplinary and experiential perspectives, including early childhood education, child development, behavior change, and digital technology. We also worked with the University of British Columbia’s Child Care Services and the City of Richmond to access childcare sites used as the backdrop for the videos in the intervention
  Conducted a literature review on early childhood educator perceptions of outdoor play in the early learning and childcare center setting
  Conducted a needs assessment of the target population through 5 focus groups with early childhood educators, administrators, and licensing officers to explore their perceived key issues, needs, and challenges that required intervention
  Developed a logic model of the problem. The literature review and focus group data informed identification of the problem and determinants influencing the problem
  Established intervention goals. The logic model helped identify the goals and targets for the intervention that related to determinants that were amenable to change

- Step 2: Intervention objectives and outcomes
  Developed a logic model of change to identify what needs to change and for whom. This linked the behavioral and environmental change objectives to specific outcomes that helped meet the intervention objectives

- Step 3: Intervention design
  Selected theory- and evidence-based behavior change methods. Social cognitive theory formed the theoretical basis for our choice of behavior change techniques
  Selected optimal applications and technology to deliver the intervention. The OutsidePlay web-based intervention had proven effective in supporting parents’ attitude and behavior changes [25]. The participants in the focus groups stressed the importance of easy access to any resource and that a web-based intervention can provide an efficient, convenient, and inexpensive means to support behavior change with broad reach [29]
  Developed a mock-up of the intervention grounded in social cognitive theory
  Tested the intervention through 12 cognitive interviews with early childhood educators to assess their perceptions of the intervention

- Step 4: Intervention production
  Refined the intervention based on cognitive interview findings and partners’ and collaborators’ feedback
  Engaged in full production and finalizing of the intervention

- Step 5: Intervention implementation plan
  Developed the intervention implementation and mobilization plan. This sought to ensure that target audiences would be aware of, and motivated to engage with, the intervention

- Step 6: Intervention evaluation
  Identified evaluation objectives and methods
  Will conduct single-blind randomized controlled trial to test the efficacy of the intervention

We obtained ethics approval from the University of British Columbia and Children’s and Women’s Health Centre of British Columbia Research Ethics Board (H19-01230; H19-03644). The health risks of the intervention are negligible. The potential benefits are that participants learn more about the importance of children’s outdoor play and engage in desirable changes in their practice that allow children more opportunities for high-quality outdoor play.

### Participant Recruitment and Eligibility Criteria

The inclusion criteria for the focus groups and individual cognitive interview participants included the following:

1. Being aged ≥19 years.
2. Being an ECE, ELCC administrator, early childhood education faculty or student, or licensing officer in Metro Vancouver.
3. Being able to speak, read, and understand English.

We recruited participants through a variety of means that we have successfully used in past research. These included using social media such as Facebook and Facebook advertisements, mass emails through our partner networks, and snowball methods.

The inclusion criteria for the RCT participants will include the following:

1. Being aged ≥19 years.
2. Currently working as an ECE or ELCC administrator in Canada.
3. Being able to speak, read, and understand English.

Given that the study will be conducted entirely on the web, computer and internet literacy is in fact an implicit eligibility criterion. Eligible participants will provide consent on the web by selecting checkboxes. Once done, a copy of the completed consent form will be made available for download. The enrolled participants will be invited to fill in the baseline measures in REDCap (Research Electronic Data Capture) and enter their email address to which a unique link to their intervention or control materials will be sent. We will use the participant email address only to administer web-based baseline and follow-up measures and will not share this information with the researchers who will be conducting the analysis. The participant email addresses will be also used to prevent multiple trial entries. The RCT study will be a purely web-based trial where outcomes will be self-assessed through questionnaires.

We will aim to recruit 324 ECEs and ELCC administrators through a variety of web-based means, including Facebook advertisements and mass emails through our partner networks. The institutional affiliations (ie, the University of British Columbia and the BC Children’s Hospital Research Institute) will be displayed on the consent form and throughout the web-based RCT survey as well as in the OutsidePlay intervention. There will be no human involvement unless the participants have inquiries or report technical issues. RCT participants who have any questions or feedback to share can contact us by email or phone, details of which will be provided in the trial and the OutsidePlay intervention.

The participants will not need to pay to participate in the study; however, they will need to be working as ECEs as stated in the eligibility criteria. As for participant remuneration, a nominal honorarium of $50 was paid to each focus group interview participant; in addition, they received a professional development certificate for their attendance. The focus group interview participants were paid cash at the end of the focus group in which they participated. The individual cognitive interview participants also received US $40 each for their participation. The RCT participants will receive US $24 at baseline and US $20 at each of the 2 follow-ups, totaling US $64. The RCT participant honoraria will be processed through electronic transfer using participant email addresses. In addition, the RCT participants allocated to the intervention group who complete the OutsidePlay intervention will receive a professional development certificate for 100 minutes.

### Trial Status

Participant recruitment began on December 1, 2020, and was completed by March 15, 2021. Follow-up data collection was ongoing through July 2021.

## Results

### IM Step 1: Understanding the Problem

#### Needs Assessment Focus Groups

A literature review examined current discourses on ECE perceptions of outdoor play at ELCCs, focused on identifying the perceived barriers and facilitators. This was performed to inform the intervention and draft the semistructured focus group interview questions for the needs assessment (Multimedia Appendix 1). The focus group interviews explored ECEs’ and ELCC administrators’ perceived key issues, needs, and challenges that required intervention (eg, what challenges do you encounter around supporting children’s outdoor play? What would help support you in that role?).

We conducted 5 focus group interviews with 40 ECEs, ELCC administrators, ECE faculty and students, and licensing officers in the summer of 2019. Of the 40 participants, 40 (100%) were women, which clearly reflected the dominant proportion of women in the ECE-related field [30,31], and 33 (83%) were ECEs, although many held >one role. After obtaining the participants’ consent and demographic information, a member of our research team (MB, FM, or MZ) facilitated the session, whereas 2 others (FM, MZ, or TC) took notes and sought clarifications when necessary. All research team members completed field notes to document their reflections after the focus group. Each focus group lasted between 60 and 90 minutes. The focus group interviews were transcribed verbatim and analyzed using thematic analysis methods [32].

#### Focus Group Results

A thematic analysis was conducted using a socioecological framework [33] to consider the identified needs and challenges at the individual, interpersonal, organizational, and policy levels and the interconnections and interactions among these levels. The complete focus group methods and findings have been submitted for publication elsewhere, and a brief summary of the findings is provided below.

At an individual level, all the participants agreed that outdoor play is an important part of childhood and that they could play an important role as facilitators. However, many acknowledged fear and anxiety regarding the potential for injury. The participants had a range of different beliefs about children’s general competence to manage their own risks. They were also concerned about differing levels of risk tolerance among ECE colleagues, administrators, licensing officers, and parents, which made them apprehensive of their actions being criticized and viewed as negligent. These beliefs, which were often linked to their own childhood and their previous experiences as ECEs, either facilitated or impeded their support for children’s outdoor play.

At an interpersonal level, many participants discussed the importance of building positive relationships with children, colleagues, parents, and licensing officers. Positive and continual communication was deemed fundamental to boosting ECEs’ and ELCC administrators’ self-confidence and agency in facilitating and promoting children’s outdoor play. In particular, the participants proposed that understanding and embracing their colleagues’ differing levels of risk tolerance could create a comfortable and flexible space to foster children’s outdoor play, as well as opportunities for ECEs to build their risk tolerance.

At an organizational level, the participants discussed the quality and availability of the outdoor space at their centers. Some ECEs were concerned that their outdoor play spaces were inadequate, which limited their use of these spaces. At the broader community and societal level, the participants discussed their views of existing licensing regulations. Some found them too vague, whereas others cautioned that more prescriptive guidelines would be excessively restrictive.

In response to questions regarding what the participants perceived would be helpful in supporting their role in facilitating children’s outdoor play, all agreed that an intervention was needed that could educate ECEs and ELCC administrators about the importance of outdoor play and help them to manage their fears around the risk involved, as well as support children’s risk taking.

However, the participants cautioned that the support would need to be flexible enough to suit ECEs and ELCC administrators with differing levels of knowledge, experience, and tolerance for children’s outdoor play. A specific recommendation was to build the intervention so as to make it adaptable for users to use as little or as much of the intervention as they would like, enabling them to go at their own pace and return to the intervention as many times as they would like. The participants also suggested that it would be ideal to keep the duration of the intervention to up to an hour, include children of different age groups, and use relatable images (ie, nonideal conditions that included artificial surfaces, urban environments, and adverse weather conditions). The participants stressed the importance of packaging the intervention using plain language—suitable for newcomers to Canada—while acknowledging diverse cultural and demographical contexts, as well as a positive and encouraging ethos that recognizes the ECE as the expert and person best positioned to support quality outdoor play.

#### Logic Model of the Problem

**Figure 1:**
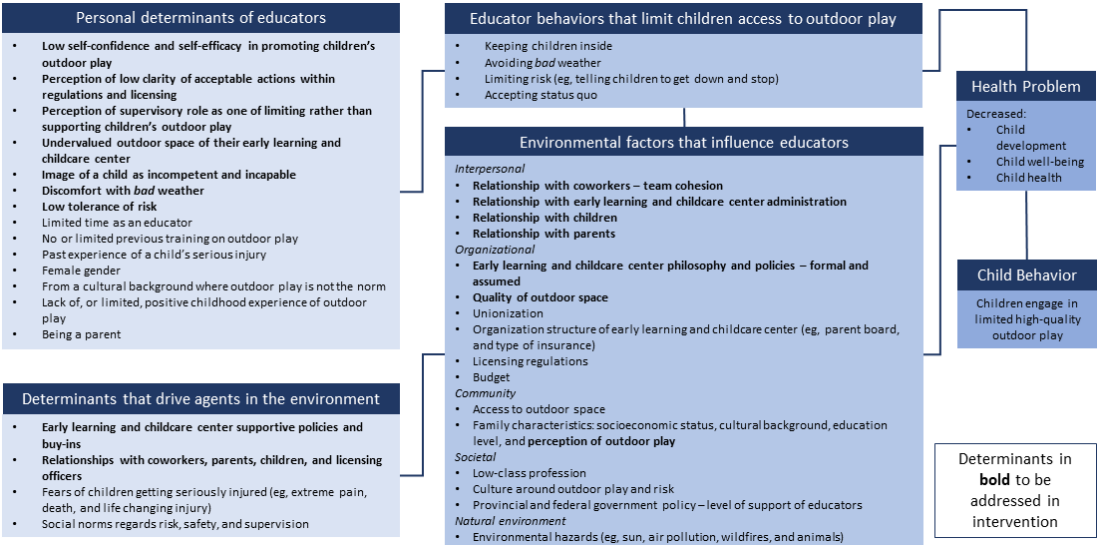
Logic model of the problem.

The literature review and focus group findings were translated into a logic model of the problem for the intervention (Figure 1). It yielded a clear set of determinants that could be addressed (or not) in the intervention, focusing on personal determinants within the individual (behavior change) and interpersonal levels (environment change).

#### Intervention Goal

The intervention goal is to support ECEs and ELCC administrators to increase children’s access to regular, sustained, and high-quality outdoor play at ELCCs.

### IM Step 2: Intervention Outcomes and Objectives

**Figure 2:**
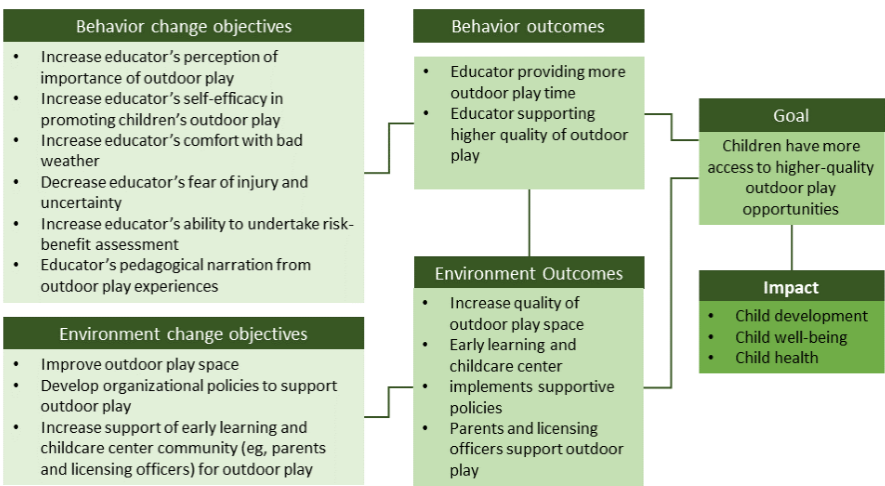
Logic model of change.

The logic model of change identified behavior and environment change objectives and outcomes for the intervention (Figure 2).

### IM Step 3: Intervention Design

#### Theoretical Underpinning

We used SCT [26] as a theoretical framework to guide the intervention design. SCT conceives that individuals are most motivated to act when they have high self-efficacy and dissatisfaction and believe that a change in behavior will lead to the desired outcome. More specifically, SCT includes elements such as self-efficacy (“I am capable of providing children at my center more opportunities for outdoor play”), outcome expectations (“Risky play will benefit children”), and self-evaluated dissatisfaction (“My fears are potentially harming children”).

Underpinned by the notion that behavioral patterns are primarily determined by their social and cultural contexts, the taxonomy of BCT provides an array of strategies that help to change individuals’ behavior [34]. To efficiently use this method in the OutsidePlay web-based intervention, each behavior and environment change objective was paired with a technique that can target the given determinant, which can be translated into a practical application in the target population’s contexts [35]. The selected BCTs aligned with the behavior and environment objectives, providing a practical and specific evidence-based approach.

#### Intervention Content

A mock-up of the intervention was developed, consisting of the homepage and 3 chapters that guide users through a personalized journey: a brief introduction about children’s outdoor play, self-reflection, interactive video scenarios, and goal setting. Table 1 summarizes the intervention content, the SCT constructs that were addressed, and the BCTs used for each element of the intervention. The interfaces of the intervention are available in Multimedia Appendix 2. The full intervention can be viewed on the OutsidePlay website [36].

**Table 1:** Intervention content, social cognitive theory constructs addressed, and behavior change techniques used.

Intervention	Description	Social cognitive theory construct	Behavior change technique^a^
Homepage: Introduction	Introductory video on the benefits of outdoor play and introducing the intervention Definition of outdoor and risky play and why it is important Description of the intervention components Logos of study partners	Outcome expectations Knowledge	5.1 Information about health consequences 5.3 Information about social consequences 5.6 Information about emotional consequences 9.1 Credible source
Chapter 1: Reflection	Introductory video to chapter 1 Self-reflection questions about the user’s own childhood play experience: Who were you with? Where were you? What were you feeling? Imagine the sounds, sights, and smells you were experiencing Were you inside or outside? Were you taking risks? What was your favourite thing to do? What did you get out of it? How did this experience influence you? Questions about the user’s ELCCb How do children currently play at your center? How would you like children to be able to play at your center? Finding the user’s whys What is the one main reason why you want to support children’s outdoor play opportunities? How do you support children’s outdoor play at your center? What gets in your way the most in supporting children’s outdoor play?	Outcome expectations Knowledge Barriers and opportunities	6.2 Social comparison 13.2 Framing or reframing 13.3 Incompatible beliefs
Chapter 2: Six Interactive Video Scenarios	Introductory video to chapter 2 Six interactive video scenarios Communicating with parents and caregivers Rough-and-tumble play Play at heights Conflict resolution Play with loose parts Play at speed	Outcome expectations Knowledge Observational learning Barriers and opportunities Self-efficacy Behavioral skills	1.2 Problem solving 4.1 Instruction on how to perform the behavior 5.1 Information about health consequences 5.3 Information about social and environmental consequences 5.6 Information about emotional consequences 6.1 Demonstration of the behavior 6.2 Social comparison 9.1 Credible source 9.3 Comparative imagining of future outcomes 13.2 Framing or reframing
Chapter 3: Creating Your Plan	Introductory video to chapter 3 Guide the user to establish a manageable goal to support children’s outdoor play at their ELCC: What is one thing that you can do to support children’s outdoor play? Invite the user to set a timeline for the goal	Outcome expectations Self-efficacy Behavioral skills Intentions	1.1 Goal setting (behavior) 1.2 Problem solving 1.3 Goal setting (outcome) 1.4 Action planning

^a^The behavior change technique numbers in this column correspond to the numbering in the behavior change technique taxonomy described in the study by Michie et al [34].

^b^ELCC: early learning and childcare center.

The Homepage greets users with a video introducing the OutsidePlay web-based intervention. It then invites users to take part in a personalized journey to learn more about how to support outdoor play and set a plan to reach their goal. The Homepage then unpacks essential information about children’s outdoor play. It covers what is outdoor play and why it is important for children, as well as the common challenges and barriers that ECEs and ELCC administrators encounter. The Homepage was built considering the BCTs of information about health, social, and emotional consequences and credible sources.

Chapter 1 is designed to guide users to find their reasons for why they want to promote children’s outdoor play at their center. It invites users to think about their own childhood (where they played and whom they played with) and how they felt when they were playing outside and taking risks. This brings in users’ private realms to have them more invested in the topic and equipped with perspectives other than those of ECEs or ELCC administrators. This exercise is to prepare users to compare and contrast their own outdoor play experiences with those of the children at their center. This affords an opportunity for users to critically assess why it is important for them to support children’s outdoor play and reflect on their role. Users are then guided to consider the barriers and challenges they perceive in supporting outdoor play. In this chapter, the intervention uses BCTs of social comparison, framing or reframing, and incompatible beliefs.

Chapter 2 presents interactive video scenarios with animated characters in real-life ELCC outdoor space backgrounds. Initially, 8 scenarios were proposed: (1) communicating with parents and caregivers, (2) rough-and-tumble play, (3) play at heights, (4) conflict resolution, (5) play with loose parts, (6) play at speed, (7) play with a chance of getting lost, and (8) play near dangerous elements. These scenarios exhibit different situations that could happen in ELCC contexts based on the idea of outdoor risky play [37]. These scenarios are meant to address the barriers and challenges that users had identified in chapter 1 and offer ways to manage them. In this chapter, the OutsidePlay web-based intervention uses the BCTs of problem solving; instruction on how to perform the behavior; information about health, social, environmental, and emotional consequences; demonstration of the behavior; social comparison; credible source; comparative imagining of future outcomes; and framing or reframing to enhance their self-efficacy and outcome expectation.

Most of the scenarios embed the risk-benefit assessment process [38,39] to guide users’ ways of thinking and decision-making. The general ethos is that children are competent and capable, and ECEs and ELCC administrators are best positioned to support children’s outdoor play. This ethos underlines that children need to play freely and learn from their own efforts and mistakes, while taking responsibility for keeping themselves and others safe [38,39].

**Figure 3:**
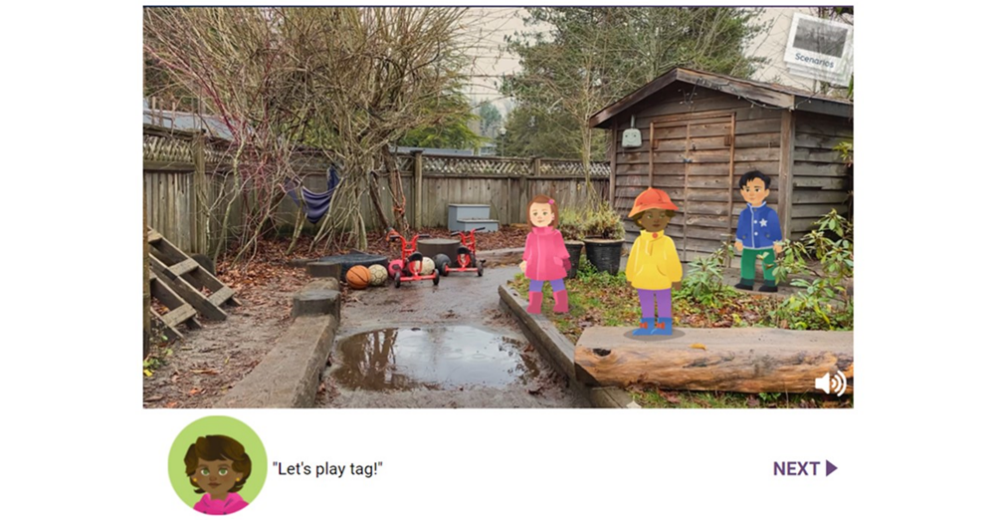
Image of the baseline story of the Play at Speed scenario.

In each scenario, users are presented with a baseline story. For instance, the play-at-speed scenario begins with children playing tag on slippery ground (Figure 3). An ECE then appears and asks users what they should do and presents 2 possible choices based on the risk-benefit assessment process, for example, (1) “It’s wet and muddy, let’s play indoors” (active intervention) or (2) “Let the children keep playing” (open observation). The rest of the scenario plays out according to the choice users make, followed by a debrief video by an ECE summating the key takeaways and providing practical guidance for users. As the final step in chapter 2, users are invited to think about the most important message that they inferred from the given scenario.

In chapter 3, with the learning imbibed from chapters 1 and 2, users are invited to think of a concrete and achievable goal and create a plan to accomplish it. It is a personalized journey, where they can carefully consider their barriers and challenges in providing children outdoor play at their center while focusing on their whys. They have the options to print out or email themselves their complete journey map, including their goal and the timeline. The OutsidePlay web-based intervention can take up to 100 minutes to complete, depending on participants’ movements through each chapter.

#### Testing the Intervention With the Target Population

We conducted 12 individual cognitive interviews to test the intervention mock-up with the target audience. This involved observing the participants as they navigated the content and probing their thoughts and reactions. The individual cognitive interview participants were all women (12/12, 100%), many of whom held more than one role, including ECE (8/12, 67%), ELCC administrator (2/12, 17%), licensing officer (3/12, 25%), and ECE student (1/12, 8%), and had been working in the field for a mean of 17.9 (SD 9.23) years. After obtaining participant consent and demographic information, the full mock-up of the OutsidePlay web-based intervention was presented on PowerPoint (Microsoft Corporation) slides. As the primary purpose of the individual cognitive interview was to assess the resonance of the intervention with the target population, we focused on gathering practical feedback (eg, What do you think about the format of this intervention? Do you find this session engaging? What did you expect to find or learn before you started using the intervention? and Would you use it as a resource for children’s outdoor play?). Each cognitive interview lasted 60-90 minutes.

#### Cognitive Interview Results

Of the 12 participants, 12 (100%) agreed that an intervention on children’s outdoor play was timely, reflecting the recent growing interest in the topic of children’s outdoor play. Their feedback helped ensure the use of appropriate language within the intervention that reflected the terminology used in the current early childhood education field. Originally, 8 interactive video scenarios were proposed to be included. On the basis of the individual cognitive interview participants’ preferences, 2 were removed. The 6 scenarios that the participants found most resonant and applicable to their ELCC contexts were (1) communicating with parents and caregivers, (2) rough-and-tumble play, (3) play at heights, (4) conflict resolution, (5) play with loose parts, and (6) play at high speed. Play with a chance of getting lost and play near dangerous elements did not make the final list because most of the participants did not find them relevant to their practice. For example, most of them assessed that their ELCC’s outdoor play space would not allow children to get lost (eg, fenced-in facilities or situated in a high-rise building), and losing a child on a field trip was perceived as very unlikely. As for play near dangerous elements, we proposed a scenario that involved children taking their boots off and playing in a mud puddle. Although we originally considered it a modest example of the dangerous natural element aspect of the scenario, the participants did not find this scenario relatable and, instead, suggested adding the muddy and slippery element into the play-at-high-speed scenario.

In addition, based on their feedback, we enhanced some of the answer options for the self-reflection questions in chapter 1 and used plain language as much as possible with an encouraging tone, acknowledging users with varying degrees of comfort and knowledge in outdoor play. For example, we made a series of short videos that premised each question and acknowledged the different positions and backgrounds that users might come from. The intention was to create a safe place for users to reflect on the question.

### IM Step 6: Intervention Evaluation Plan

#### Hypotheses

We will assess the efficacy of the intervention to increase ECEs’ and ELCC administrators’ tolerance for children’s outdoor play (primary outcome) and attain a behavior change goal related to providing outdoor play opportunities for children at their center (secondary outcome). We hypothesize as follows:

1. The participants completing the OutsidePlay web-based intervention will have a significantly greater increase of tolerance of risk in play than those in the control group.
2. A greater proportion of the participants completing the OutsidePlay web-based intervention will attain their behavior change goal than those in the control group.

#### RCT Design

The study will use a single-blind (researchers and outcome assessors), two-parallel-group RCT design to determine the superior efficacy of the intervention over the control group. The trial has been registered on ClinicalTrials.gov with the US National Institute of Health’s Protocol Registration and Results System [40] (NCT04624932), which was released on April 26, 2021.

Information on the study will be available for the potential participants’ review on the web. It will detail the entire study procedure, including the consenting, randomization, and follow-up process. When the eligible participants provide consent to participate, they will be invited to complete the baseline survey, including a demographic questionnaire and survey measurements. Next, each participant will be randomized to 1 of the 2 groups by the REDCap electronic data capture intervention [41] hosted at the BC Children’s Hospital Research Institute. The randomization list will be generated beforehand by the Sealed Envelope service (Sealed Envelope Ltd) using randomized permuted blocks of sizes 4, 6, and 8. The list will then be transferred to REDCap. The groups include (1) the control and (2) the OutsidePlay web-based intervention. The participants may assume which intervention is the *intervention of interest* based on the details of the 2 groups involved in the RCT in the consent form: control (eg, generic information) and intervention (eg, web-based tool). The participants will have equal likelihood of assignment to each group using a basic (rather than stratified) randomization method. They will not be blinded to allocation because the nature of the intervention does not allow it. There will be no outcome assessors because it will be exclusively on the web. Allocation will be concealed to the researchers at participant assignment.

We initially considered stratifying randomization by different characteristics. However, ELCCs in Canada are very diverse, and not all of them would necessarily influence access to higher-quality outdoor play. From the literature, key characteristics include the perceptions of the ECEs toward outdoor play as well as the quality of the available outdoor play environments. These can vary widely, regardless of the region, urban or rural location, or other site characteristics. Given that our intervention is designed to influence ECE perceptions, stratification according to this characteristic was not appropriate.

#### RCT Sample Size Consideration

The Teacher Tolerance of Risk in Play Scale (T-TRiPS) [42] will be our main study outcome. The T-TRiPS is a 25-item measure with dichotomous yes or no responses on items that reflect the 6 categories of risky play in the study by Sandseter [1] (great heights, high speed, dangerous tools, dangerous elements, rough-and-tumble, and disappear or get lost). Sample items include, “Would you let your students climb as high as they wanted in a tree or another surface?” and “Do you wait to see how well your students manage challenges before getting involved?” Possible scores range from –5 to 5, with higher scores reflecting more risk tolerance; in previous research, the scores ranged from –4.09 to 4.56 [42]. The T-TRiPS is a modified version of the TRiPS for parents [43] to measure teachers’ perceptions of risk. The T-TRiPS has been psychometrically validated [42], and we will administer it strictly on the web through REDCap where participants will input their responses directly.

With a sample size of 206 ECEs in total, a linear mixed model examining the impact of intervention compared with control, including an interaction with time, will have 80% power at 0.05 level of significance to detect a difference of 0.75 between the intervention and control groups when the SD is 1.82 and the correlation value between repeated observations is 0.75. From our previous work [24,25], we anticipate requiring 324 complete baseline assessments among ECEs and ELCC administrators who will then be randomized into the 2 groups. Specifically, we are assuming a retention rate of 74.7% (242/324) at our first assessment and a retention rate of 85.1% (206/242) at our second assessment, which would result in a final sample of 206 ECEs, corresponding to 103 in each group.

#### Interventions

The participants in the control group will be provided with a PDF copy of the Position Statement on Active Outdoor Play, which includes information on research and recommendations for action regarding children’s outdoor play [44,45]. This 4-page document was developed by a cross-sectoral consortium of researchers, practitioners, and stakeholders to provide recommendations for parents, educators, health professionals, administrators, and various levels of government to address the barriers to children’s outdoor play. This PDF will be delivered on the web through REDCap. The participants in the web-based intervention group will be provided with a link to the intervention [36].

#### RCT Measurement Occasions and Follow-ups

The participants will complete a questionnaire package at 3 time points: baseline, before the intervention; 1 week after the intervention; and 3 months after the intervention. Long-term change is unlikely if participants do not make initial changes; thus, the 1-week postintervention follow-up was selected to assess short-term efficacy, while still providing participants sufficient time to make their initial planned changes. The 3-month postintervention follow-up will assess long-term efficacy once the participants have had 3 months to reflect on the intervention and implement change. Survey data will be collected and managed using REDCap [41]. Baseline data collected will include sociodemographic data: sex, age, language spoken most often at home, province of employment, role, and length of time in the early childhood education field. The participants will also complete measures to assess primary and secondary outcomes at each time point. We are anticipating completing the data collection by the end of summer 2021.

#### RCT Outcome Measures

The primary outcome measure is change in the participants’ T-TRiPS score. We expect an increase. The secondary outcome measure is self-reported behavior change on attaining the goal that the participants set for themselves upon completion of the allocated intervention. At baseline, the participating ECEs will be invited to think about what they could do to give children at their center more opportunities for outdoor play and to set a specific and realistic goal for themselves. This goal will be used at the 2 follow-ups to assess their goal attainment. The participants will be asked to report if they have attained their goal (ie, yes or no) at 1 week and then 3 months after the intervention.

#### Adherence to Intervention

Adherence to the OutsidePlay web-based intervention will be measured and verified by an automated system that will archive complete participant journey maps (refer to step 3).

#### Data Management

Data will be entered by the participants directly into REDCap, which is hosted on a secure, firewall-protected server at the BC Children’s Hospital Research Institute. The database is password protected and only accessible by responsible staff members. REDCap maintains an audit trail that captures all user activity, including data manipulation and export. Exported data will be stored on a secure, firewall-protected server at the BC Children’s Hospital Research Institute in a password-protected file only accessible by responsible staff members.

The participants’ confidentiality will be highly respected, and each participant will be assigned a unique study number in the trial. This number will not include any personal identifiable information. A contact (ie, the University of British Columbia Office of Research Ethics) to report concerns about the rights of the research participants will be provided. This is to ensure that the participants have access to support if the trial was harmful to them.

#### RCT Statistical Analyses

All participants allocated to 1 of the 2 groups will be included in the analyses, regardless of deviation from the protocol, missed follow-up observations, or withdrawal. All baseline characteristics will be summarized by means and SDs or frequencies and percentages as appropriate by intervention group. T-TRiPS data will be visualized in the form of box plots by intervention group and time. To test our hypothesis that the ECEs completing the intervention will have a significantly greater increase of tolerance of risk in play, linear mixed effects models will be built to assess the relationship between intervention group and T-TRiPS score. Time will be included in the model as a categorical variable, and the baseline T-TRiPS score as well as an interaction term between time and intervention will be included to explore the possibility that the impact of the intervention diminishes over time. Intent-to-treat analysis of T-TRiPS scores will use last observation carried forward as the method of imputation. To test our hypothesis that a greater proportion of the participants completing the intervention will attain their behavior change goal than those in the control group (secondary outcome measure), the 3-month postintervention behavior change goal outcome (yes or no) will be the primary outcome of interest, and we will use logistic regression analysis. Model diagnostics will be run to test modeling assumptions. We will not perform any additional analyses such as subgroup or adjusted analyses.

#### Quality Assurance and Monitoring

A written standard operating procedure and researcher protocol manual will be used for staff training for all study procedures to ensure data quality and consistent application of the study protocols. The state of recruitment, data completeness, control of correct randomization, and allocation of participants will be regularly verified. In all, 3 sets of automated reminders will be deployed to the participants’ email addresses if the baseline survey is not completed within 24, 48, and 60 hours. The participants will also receive 3 sets of automated reminders (eg, 1 each day for 3 days) through email at the 1-week and 3-month postintervention follow-ups, provided that they have completed their baseline requirement. Any deviations from the expected standards will be reported to, and discussed with, the study manager. Any protocol modifications will be reported to the University of British Columbia and Children’s and Women’s Health Centre of British Columbia Research Ethics Board, as well as the US National Institute of Health’s Protocol Registration and Results System.

### IM Step 4: Intervention Production

After making modifications to the intervention based on the cognitive interview participants’ feedback and expert advice from partners and collaborators, we finalized the mock-up and moved into full production, developing user interface, media, and subsequently assembling the platform in its entirety. A series of beta-testing sessions was conducted with 9 participants: 5 (56%) through individual cognitive interviews and 4 (44%) who tested the intervention at their own convenient time and shared feedback through email. The aim of beta testing was to finesse the OutsidePlay web-based intervention itself (eg, fix bugs and glitches) and to prepare for the RCT in step 6.

### IM Step 5: Intervention Implementation Plan

Consistent with the BCT credible source [34], a partnership with trusted organizations within the ECE sector in Canada is critical to the implementation of the intervention once it has been evaluated. We have extensive relationships in the ECE sector in Canada and sought to increase the persuasiveness of the message in the OutsidePlay intervention by using our target audience’s pre-existing and trusted communication channels. We identified >600 relevant childcare programs and centers, postsecondary institutions, childcare resources and referral centers, and government departments across Canadian provinces to assist with deployment of the intervention once it has been evaluated.

We developed marketing materials suitable for the organizations and target audience, including the introductory video on the OutsidePlay web-based intervention that could be posted separately from the platform. As described in Table 1, the introductory video addresses the SCT constructs of outcome expectations and knowledge through the BCTs information about consequences (health, social, and emotional) from a credible source (university and children’s hospital). Furthermore, we developed 5 infographics depicting various key messages of the intervention that included a quick response code directing users to the intervention [36] (Multimedia Appendix 3).

Consistent with the BCT rewarding completion, we contacted ECE registries in Canadian provinces to register the intervention as a workshop, where applicable, to provide professional development certificates for users who completed the intervention. This option was available for ECEs for 6 of the 10 Canadian provinces. Furthermore, many registries agreed to assist with the RCT participant recruitment and promote the intervention to their members once the RCT results were finalized. The intervention was soft launched on December 1, 2020 [36]. It will be officially launched upon completion of the RCT analyses. The intervention content will be frozen during the RCT.

## Discussion

### Outdoor Play Within ELCCs

The importance of outdoor play to children’s health development and outcomes is clear [1-3]; yet, there are many barriers to ensuring daily opportunities for high-quality outdoor play. ELCCs can be critical settings for outdoor play opportunities and can help reduce inequities in access to outdoor play. Many ELCCs struggle to provide regular and high-quality outdoor play opportunities, citing multiple perceived barriers [15,16]. In Canada, there are few interventions to increase outdoor play opportunities for children in ELCCs; most of the existing ones consist of professional development workshops of varying length and quality. To our knowledge, no interventions have been developed that are grounded in health behavior change theory and techniques and none that have been evaluated through an RCT. Influenced by our previous success in developing an effective intervention for parents [25], we designed the OutsidePlay intervention to shift ELCC stakeholders’ perceptions and practices to support outdoor play within ELCCs.

### Strengths and Limitations

Guided by the systematic IM process and using BCTs grounded in SCT, the OutsidePlay web-based intervention represents a novel, evidence-based, and rigorously designed tool to support change. The IM process enabled considering and addressing the target population’s key issues, needs, and challenges in the context of providing children high-quality outdoor play in the ELCC setting. More specifically, the intervention design and development followed an organic process that involved collaboration among researchers, practitioners, and digital technology experts, with regular consultation with the target population. This inclusion of various stakeholders from the outset enabled the development of content that was relatable, acceptable, and engaging, using the preferred modality and user-friendly media.

Furthermore, the web-based format reduces barriers to uptake by allowing for widespread and free access. Web-based delivery also made it possible for users to use the intervention at their convenience and to return to the intervention, picking up from where they left off. The RCT will evaluate the efficacy of the intervention, providing necessary evidence to inform the mobilization of the intervention and widespread efforts to support children’s outdoor play. We expect that a routine application setting will be slightly different than the protocol used in the trial because it will not involve reminders and payment upon completion of the intervention. However, users who use the intervention in a nontrial setting will still be able to receive a professional development certificate for 100 minutes.

The primary study limitation is that the accessibility of the intervention is not always guaranteed because of the system and bandwidth requirements of the content in the intervention (ie, high-resolution media). This issue was well known from our previous study, and we designed the intervention to adjust the quality of videos and images based on each user’s internet bandwidth. However, this did not solve the problem caused by users accessing the intervention from old or incompatible devices. Likewise, although we attempted to ensure that the intervention was as user-friendly and easy to navigate as possible, a minimum level of computer literacy was necessary to use it. We recognize that internet access and computer literacy are issues with the potential to increase inequities and that they require further careful consideration.

Second, because of the nature of the intervention, the participants could not be blinded, which is a typical limitation in eHealth trials. During the consenting process, the participants will be informed that there will be 2 groups in the trial (ie, control and intervention). The participants may be able to determine the group they are assigned to based on the differences in time commitment.

Another limitation stems from the study samples included in step 1 (focus groups) and step 3 (cognitive interviews). Most of the interview participants resided in the Metro Vancouver area (urban or suburban) in British Columbia, Canada. Hence, key issues, needs, and challenges pertinent to other Canadian provinces, let alone other countries, may not be reflected in the intervention. For example, the Metro Vancouver area has a milder climate than many other parts of Canada. Therefore, specific cold weather–related issues (eg, snow and freezing rain) were not prominent in our interviews.

### Conclusions

The OutsidePlay web-based intervention guides users through a personalized journey that is grounded in behavior change theory and techniques. If effective, this relatively low-cost, easily accessible intervention may have the potential to address ECEs’ and ELCC administrators’ perceived challenges and needs in promoting and accommodating children’s outdoor play in ELCC settings, thereby supporting high-quality early childhood education.

## Appendix

Multimedia Appendix 1 Focus Group Interview Questions.

Multimedia Appendix 2 Interfaces of OutsidePlay Intervention.

Multimedia Appendix 3 Infographics.

## Abbreviations

BCT: behavior change technique
ECE: early childhood educator
ELCC: early learning and childcare center
IM: intervention mapping
RCT: randomized controlled trial
REDCap: Research Electronic Data Capture
SCT: social cognitive theory
T-TRiPS: Teacher Tolerance of Risk in Play Scale
