# Supplementary material for: A Web-Based Risk-Reframing Intervention to Influence Early Childhood Educators’ Attitudes and Supportive Behaviors Toward Outdoor Play: Protocol for the OutsidePlay Study Randomized Controlled Trial
Source: JMIR Res Protoc. 2021 Nov 18;10(11):e31041. doi: 10.2196/31041 (PMC8663711; doi:10.2196/31041)
Supplement: Multimedia Appendix 1 [file resprot_v10i11e31041_app1.pdf]

Early Childhood Outside (ECO) Practice Tool

Focus Group Interview Questions

1. What does outdoor play mean to you?
2. What does outdoor play look like in your centre?
3. What challenges do you encounter around supporting outdoor play?
    a. Probes: What are children allowed to do and not allowed to do? How do you decide?
4. Tell me about a time when you let a child play in an unexpected way. What helped you let go? Why did you see it as important to let it happen?
5. Tell me about a time when outdoor play transformed a child's experience?
6. Tell me about a time when your observation of outdoor play transformed your experience as an early childhood educator?
    a. Probes: What did you learn from this? *or* How did it shape your practice/approach as an early childhood educator?
7. What do you see as your role in supporting outdoor play?
8. What would help support you in that role?
    a. Probe: What kind of support would be helpful from your colleagues/your centre director/parents…etc.
9. What tool would be helpful in supporting you?
    a. What kinds of elements would you want to see in an online tool?
        i. Probe: Education on importance of outdoor play and risk taking in play?
        ii. Probe: Developing strategies for change?
    b. Would it be for people like you in your role? Or some other role (e.g., director, licensing officer)?
