# Supplementary material for: A Web-Based Risk-Reframing Intervention to Influence Early Childhood Educators’ Attitudes and Supportive Behaviors Toward Outdoor Play: Protocol for the OutsidePlay Study Randomized Controlled Trial
Source: JMIR Res Protoc. 2021 Nov 18;10(11):e31041. doi: 10.2196/31041 (PMC8663711; doi:10.2196/31041)
Supplement: Multimedia Appendix 3 [file resprot_v10i11e31041_app3.pdf]

TAKE A RISK

GO PLAY Outside!

WHY IS OUTDOOR PLAY IMPORTANT?

Play is basic childhood need. Outdoor play and taking risks is a necessary part of healthy play. Whether jumping in a pile of leaves, climbing a tree, or playing street hockey, children are often happiest when playing. These kinds of experiences are a lot less common for kids today. Our worries and desire to protect our kids can result in setting too many limits on them, which can interfere with healthy development.

Risky play can have many different shapes, but always involves the thrill and excitement of testing oneself and finding out what happens.

BENEFITS OF PLAY

When children play outside, they have more freedom, are more physically active, and move their bodies in different ways. The outdoors can offer a greater variety of play environments and loose parts to interact with (e.g., sticks, rocks, buckets, sand, crates)- allowing their imagination to shape their play.

WHAT ARE THE BENEFITS OF OUTDOOR PLAY?

Children need daily outdoor play opportunities for their development, physical health, and well-being.

- Only 33% of school-aged children meet recommended physical activity guidelines
- For every additional hour spent outdoors per day, children get 7 more minutes of moderate-to- vigorous physical activity, 762 more steps, and 13 less minutes of sedentary time.
- Outdoor play promotes positive feelings – joy, thrill, competence. Too little play can result in sadness, anger, boredom, anxiety.
- Outdoor play improves concentration and attention span. Too little play can make it hard to focus and learn in new activities, limits creativity, exploration.

WHY DO CHILDREN LOVE OUTDOOR PLAY?

Children are more physically active outdoors, especially in unstructured play spaces that can shift and change in response to creativity.

PLAY SPACE

"I can test my limits and boundaries"
Play at Heights
(e.g. climbing a tree)

"I can build physical competence"
Play at Speed
(e.g. running, biking sliding)

"I can develop cooperation skills and learn the principles of science"
Play with Dangerous Tools
(e.g. hammer, nails, wood)

"I can learn how to manage risks while keeping myself safe"
Play Near Dangerous Elements
(e.g. fire, stream, lake)

"I can build independence"
Play with a Chance of Getting Lost
(e.g. adventuring around forest, roaming around neighbourhood)

"I can learn how my body works and develop social skills"
Rough and Tumble Play
(e.g. wrestling)

PLAY TIME

ARE KIDS GETTING ENOUGH OUTDOOR PLAY?

MOVEMENT GUIDELINES RECOMMEND

60 MINUTES DAILY	120 MINUTES DAILY	180 MINUTES DAILY
moderate to vigorous physical activity for *5-17 year olds*	active play suggested by ParticipACTION	of any intensity for *0-4 year olds*

- ***Only 37%*** of modern day children play outside everyday and ***only 7%*** of kids under 10 are allowed to go out on their own
- Students in grades 6-10 play outdoors for an ***average of 15 minutes per day***
- ***Only 12%*** of preschoolers meet the physical activity guidelines

APPROPRIATE WEATHER GEAR

SUNNY

hat
sunglasses
1 layer
SPF 30
sunblock

RAINY

appropriate number of layers
umbrella
waterproof boots
outer layer: waterproof jacket

CHILLY

1-2 layers
outer layer: light jacket
pants: long layer
shoes: warm

COLD

warm hat
2-3 layers
outer layer: thick jacket
pants: long layer
boots: warm, waterproof

EXTREME COLD

warm hat
face mask
3+ layers, 1 insulating (wool, fleece)
outer layer: thick jacket
pants: long layer paired with long, thick socks
boots: warm, waterproof

FREEDOM

WHAT CAN YOU DO?

Try implementing a Risk-Benefit Assessment:
REFLECT BEFORE REFLEX

STOP yourself before you respond to, or stop children's play

LOOK & LISTEN to what is happening. What are the children doing?

THINK are the children managing the risks? What might be the impact of adult involvement on children's play?

ACT now you can respond in a thoughtful way, rather than by reflex

REFLECT was your action the correct one? What have you learned?

TIP : 17 SECOND RULE: WAIT 17 SECONDS BEFORE INTERVENING TO LET IT PLAY OUT

FOR MORE INFO
WWW.OUTSIDEPLAY.CA

SCAN HERE TO WATCH AN INTRO VIDEO AND LEARN MORE.

A collaborative project by
BC Children's Hospital
UBC
BC INJURY research and prevention unit
digital LAB

Generously supported by the
Canada
LAWSON FOUNDATION
fraserhealth
Better health. Best in health care.
